# Practical statistics for bioimage analysis – a guide to experimental design and data interpretation

**DOI:** 10.1242/jcs.264367

**Published:** 2026-05-27

**Authors:** Stefania Marcotti, Lina Gerontogianni, Gavin Kelly, David J. Barry

**Affiliations:** ^1^ Image Analysis Group, Crick Advanced Light Microscopy Science Technology Platform, The Francis Crick Institute, London, NW1 1AT, UK; ^2^ Randall Centre for Cell and Molecular Biophysics, King's College London, London, SE1 1UL, UK; ^3^ Bioinformatics and Biostatistics Science Technology Platform, The Francis Crick Institute, London, NW1 1AT, UK

**Keywords:** Bioimage analysis, Reproducibility, Statistics

## Abstract

Bioimage analysis is a powerful tool for investigating complex biological processes, but its robustness depends on technical precision and rigorous experimental design. In particular, the use of appropriate controls and experimental repetition is critical for drawing meaningful conclusions. However, there are times when both are inadequately applied or overlooked in favour of ‘statistical significance’, often derived from misused or misinterpreted statistical tests. In this Perspective, we reanalyse publicly available image datasets to highlight the crucial role of robust experimental design in interpreting results. Our findings underscore the importance of focusing on effect sizes and biological relevance over arbitrary statistical thresholds. We also discuss the diminishing returns of increased data collection once statistical stability has been achieved. By refining control usage and emphasising effect sizes, this Perspective offers guidance to enhance the reproducibility and robustness of research findings. We provide open access code to allow researchers to engage with the dataset, promoting better practices in experimental design and data interpretation.

## Teaser

**Summary:** You've analysed some images, but is the result significant? Is it reproducible? Through reanalysis of published image data, this Perspective offers practical guidance on experimental design and data interpretation.

## Introduction

Bioimage analysis has revolutionised our ability to quantify and interpret complex biological phenomena. However, the robustness of these analyses hinges not only on technical execution, but also on the rigour of experimental design and the correct interpretation of results. While there are many examples of well-executed studies leveraging bioimage analysis, often leading to novel biological discoveries, there are examples in the literature that fall short. These include poorly designed experiments, a lack of adequate controls, and a tendency to falsely equate biological and statistical significance. Although time pressures and resource constraints no doubt play a role, a lack of understanding of the variability and measurement noise that is inherent in biological experimentation might also be a factor. A measured difference could appear significant, but this difference could result from day-to-day, sample-to-sample or even cell-to-cell variability, rather than any meaningful biological effect. The biological effect, or effect size, is essentially the difference in the mean value of a measurement between two cell populations, sometimes referred to as the raw effect size.

Effective controls are crucial for distinguishing true biological effects from artefacts introduced by the experimental procedure. Despite this, controls are sometimes inadequately implemented or poorly chosen, leading to misinterpretation of experimental results. Furthermore, there are examples of controls being inadequately reported, or authors failing to demonstrate how the control data directly support their specific conclusion (Cobey et al., 2024).

The role of controls is frequently overshadowed by the pursuit of statistical significance, an issue widely acknowledged as a substantial contributor to reproducibility issues (Cobey et al., 2024). For example, it is not unusual for individual measurements from within the same sample to be treated as biological ‘replicates’. Often termed ‘pseudo-replication’ by statisticians, this issue is commonly encountered in bioimage analysis when measurements from individual cells drawn from the same dish or well are presented as independent data points (see Blainey et al., 2014 for a more detailed explanation). These data points are then grouped according to treatment and subjected to statistical tests in pursuit of a ‘significant’ *P*-value. Applying tests such as ANOVA to these data is inappropriate, as cells from the same sample are necessarily subjected to the same treatment and cannot generally be considered independent. These issues are often exacerbated when non-independent data points from multiple experiments are pooled, artificially inflating sample size and resulting in misleadingly low *P*-values (Lord et al., 2020). There might be specific circumstances in which very large numbers of measurements can be treated as independent, in which case frameworks have been presented elsewhere for dealing with the issue of pseudo-replication (Gómez-de-Mariscal et al., 2021).

It is also crucial to address the misconception that a larger sample size yields more reliable results. While truly independent biological replicates increase reliability by better capturing variability, collecting an excessive number of data points from a single experiment (to achieve statistical significance) is often unnecessary and can lead to a wasteful allocation of resources. Assuming data are sampled in an unbiased manner, the distribution of values within a large population can often be well represented by a relatively small sample size. Although increasing this sample size will result in greater confidence in the obtained statistical descriptors, the law of diminishing returns eventually kicks in (Krzywinski and Altman, 2013). The point at which one should cease collecting data ultimately depends on the level of confidence needed in the statistical description of a particular population, which in turn depends on the size of the effect one is attempting to detect. The importance of unbiased data acquisition should again be stressed here, as obtaining a small number of measurements from a non-representative sub-population can lead to erroneous conclusions. However, it is important to recognise that even in rigorously designed experiments with appropriate controls, ambiguity might persist and should be explicitly acknowledged.

While there are many guides on statistics for biologists, such as the excellent ‘Points of Significance’ series in Nature (Krzywinski and Altman, 2013) and the more recently published guide to statistical tests in microscopy (Zweifach, 2024), these are more general and could be perceived as somewhat abstract and difficult to apply to actual experimental results. Other resources include a practical guide to image data exploration for cell biologists (Pylvänäinen et al., 2025), which does provide some useful guidance on statistical concepts but has a stronger focus on data visualisation and exploration. Here, we present analyses of previously published image data, emphasising that (1) interpreting biological effects is challenging without appropriate controls; (2) when interpreting effect sizes, there is a diminishing return with continued data collection; and (3) interpreting the significance of a result is challenging in the absence of appropriate repetition. To quote from Fisher and Prance (1974): “No isolated experiment, however significant in itself, can suffice for the experimental demonstration of any natural phenomenon; for the ‘one chance in a million’ will undoubtedly occur […] In order to assert that a natural phenomenon is experimentally demonstrable we need, not an isolated record, but a reliable method of procedure.”

This Perspective is divided into approximately three main sections, exploring two case studies based on previously published data, which we use to inform the framework presented in the third main section. In both case studies, we explore how statistical descriptors of a population, such as the mean, median and standard deviation, change as more data points are collected, and we argue that data collection should cease once those descriptors stabilise. We then discuss the challenge of interpreting effect sizes in the absence of appropriate controls and go on to demonstrate that experimental repeats are necessary to draw firm conclusions.

### Case study overview and rationale

**Fig. 1.**
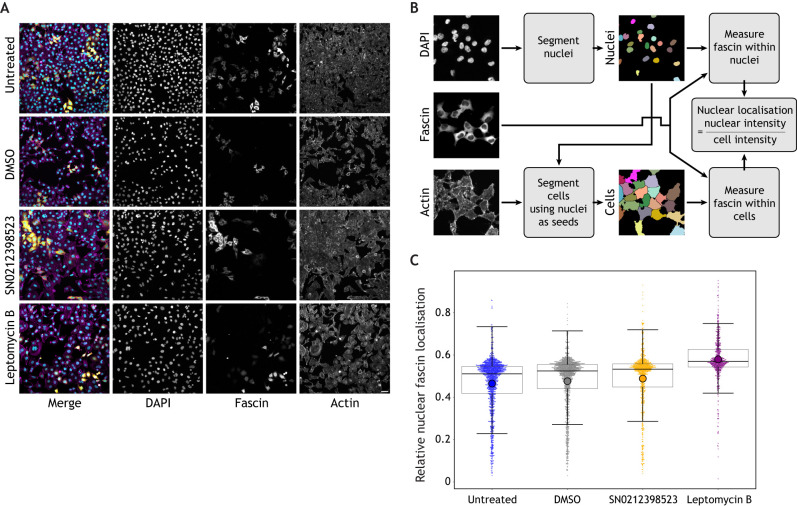
**Overview of data and analysis workflow.** (A) Images of fascin knockdown HeLa cells downloaded from the Image Data Resource (accession number IDR0139) and originally published by Lawson et al. (2022). The selected dataset includes a treated sample (pharmacological compound SN0212398523) and three controls (an untreated negative control, a DMSO-treated vehicle control and a leptomycin B-treated positive control). The images contain three channels: DAPI (nuclei, cyan), fascin (yellow) and actin (used as a cell body proxy in this work, magenta). Scale bar: 50 µm. (B) Schematic illustrating the calculation of relative nuclear intensity, implemented using CellProfiler (Stirling et al., 2021). Nuclei were detected with simple intensity thresholding, and cells were segmented using watershed segmentation, with nuclei used as seeds. See Supplementary information for more details. (C) Graphical representation of the data shown in Table 1. Each small point represents a single cell from one of four different wells (corresponding to each of the four different treatments shown) on the same multi-well plate (see Table S1). The larger dots represent the population means. Box and whisker plots are overlaid, with the height of the box, the horizontal line within the box and the whiskers representing the IQR, the median and the spread of the data excluding outliers (IQR±1.5×IQR), respectively. Images in A and B from Image Data Resource accession IDR0139 are reused under the terms of a CC BY 4.0 licence.

This study utilises two datasets, IDR0139 (Lawson et al., 2022; case study 1) and IDR0028 (Pascual-Vargas et al., 2017; case study 2), both of which are freely available from the Image Data Resource (https://idr.openmicroscopy.org; Williams et al., 2017). We chose these datasets as they meet the following desirable criteria: (1) the images are publicly available; (2) the datasets contain appropriate controls; (3) the data consist of two-dimensional images of cultured cells, labelled with nuclear and actin markers, and are thus relatively easy to analyse (Fig. 1A); and (4) it was possible to extract an intuitive and easy-to-grasp metric quantifying the proportion of a third marker in the cell nucleus. The analysis pipeline consisted of segmenting the nucleus and actin channels (where actin serves as a proxy for the cell body), quantifying the intensity of a protein of interest in both compartments, and obtaining a localisation ratio between the two to analyse nuclear accumulation. Details of all data used are listed in Table S1. To analyse the images, we composed a simple and openly available pipeline using CellProfiler (Stirling et al., 2021) to quantify the proportion of a protein of interest localised to the nuclei under different experimental conditions (Fig. 1B; see the Supplementary information for details of the analysis workflow). We have made the code used for generating the figures in this manuscript openly accessible online, and we encourage readers to engage with the data themselves and reproduce the figures using their own datasets (please see Supplementary information).

**Table 1.** Statistical properties of the data shown in Fig. 1C

Index	Treatment	Mean	Standard error of the mean	Median	25th percentile	75th percentile	Standard deviation	*n*_cells_
1	Untreated	0.465	0.013	0.511	0.418	0.547	0.131	1362
4	SN0212398523	0.488	0.017	0.533	0.448	0.558	0.140	844
5	Leptomycin B	0.579	0.024	0.569	0.543	0.626	0.108	606
8	DMSO	0.476	0.013	0.524	0.441	0.554	0.131	1299

The Index column corresponds to the index listed in Table S1 to indicate the source of the data. The metric being compared, nuclear fascin localisation, is calculated according to Eqn 1 (see Supplementary information).

Drawing conclusions about biological effects is difficult in the absence of appropriate controls and experimental replication, due to the technical variation inherent to any experiment. For example, even with the use of robotic automation, the concentration of an antibody or dye will be slightly different in different wells. Subtle manufacturing defects could cause well bottoms to vary in height or alignment across a plate, shifting the focal plane between wells – a problem that autofocus systems can mitigate but not eliminate. Both of these variables will influence the output of an image analysis pipeline, perhaps causing slight differences in the results of cell or nuclear segmentation and the resultant intensity measurements made within those regions. We demonstrate that reliable results can be obtained by analysing a relatively small number of cells from a single experiment, that appropriate controls are required to correctly interpret effect sizes and that experimental repetition is essential to confirm the reproducibility of a result.

Our approach, augmented by actual microscopy data, seeks to make these statistical concepts tangible and directly applicable for cell biologists. It is our hope that this helps to ‘demystify’ statistical analysis in the context of bioimage analysis, encouraging greater transparency and reproducibility. Additionally, we aim to inspire researchers to embrace ambiguity and uncertainty to a much greater extent.

## Case study 1 – the role of controls and sample size in interpreting effect sizes

Lawson et al. (2022) examined the nuclear localisation of fascin (an actin filament-bundling protein) following the treatment of cells with a library of pharmacological compounds (Fig. 1A). The compounds are part of AstraZeneca's annotated screening library and, as such, their function is not known (see https://openinnovation.astrazeneca.com/preclinical-research/target-identification). Here, we selected the images associated with one of these compounds, SN0212398523, for reanalysis. However, the purpose of this reanalysis was not to attempt to elucidate any biological or clinical roles of the compound; the aim here was to illustrate that, in a particular experiment, a result can be obtained with high confidence by analysing a relatively small number of cells. It is assumed that this small number of cells is representative of the broader population. This is likely a reasonable assumption in the context of cells cultured on coverslips but might be a less reasonable assumption when imaging tissue sections, for example, when more care may be needed to ensure unbiased, random sampling of the population.

Lawson et al. (2022) used HeLa cells in which the wild-type fascin was knocked down and replaced with mScarlet–fascin. We considered three channels in the images, highlighting cell nuclei (stained using DAPI), fascin and actin, with the actin channel used as a proxy for the cell body (Fig. 1B). In Table 1 and Fig. 1C, we present the results of analysing all cells in each of four different wells, corresponding to treatment with the selected compound and three experimental controls. We begin by considering these results, comparing the test treatment (SN0212398523) to each control one by one. We then examine how these results change when only a subset of cells from each well is analysed.

### Analysis of all cells in each well

#### Treated cells versus a negative control

We first estimated the effect of treating the cells with SN0212398523, relative to untreated cells (negative control; Fig. 1C). If we were to compare the mean values (±s.e.m.) for these two treatments, it would suggest that SN0212398523 caused an increase in fascin localisation in the nucleus (Table 1). However, the magnitude of this effect appeared small (0.488±0.017 in treated cells versus 0.465±0.013 in untreated cells – an increase of ∼5%), and comparisons with appropriate controls should be considered before drawing conclusions.

#### Treated cells versus a vehicle control

While simple binary comparisons between treated and untreated cells is common, a more appropriate comparison would be between drug-treated cells (the drug being dissolved in DMSO) and cells treated with DMSO alone. DMSO might introduce systematic technical variation that could influence the results. Any observed effect in SN0212398523-treated cells would likely be a combination of technical variation, a biological change caused by the active agent and random variation. Therefore, additional controls are required to conclusively determine any biological significance.

Our analysis shows that treatment with DMSO (vehicle control) led to an apparent increase in nuclear fascin localisation relative to untreated cells (0.476±0.013 versus 0.465±0.013, mean±s.e.m.; Table 1). Furthermore, nuclear fascin localisation in SN0212398523-treated cells was higher than that in DMSO-treated cells (0.488±0.017 versus 0.476±0.013, mean±s.e.m.; Table 1). These results could suggest that DMSO has a measurable effect on the nuclear localisation of fascin, but the effect of SN0212398523 is greater. However, the errors associated with the mean estimates are large relative to the measured differences. We must remain aware that apparently significant differences can stem from random variation rather than biological effects. Indeed, these differences can be statistically significant, but this does not guarantee biological significance.

#### Treated cells versus a positive control

Leptomycin B inhibits nuclear export (Lawson et al., 2022), which should result in the accumulation of fascin in the nucleus, thus providing a useful positive control. Quantifying the proportion of fascin localised in the nucleus in leptomycin B-treated cells, we find that the mean value is much larger than in all other treatments (Table 1). This helps to put the other measured differences in context. For example, the difference in nuclear fascin localisation between leptomycin B-treated cells (0*.*579±0.024) and SN0212398523-treated cells (0*.*488±0.017) is 0*.*091±0.041 (mean±s.e.m.), several times greater than the difference between SN0212398523-treated cells and DMSO-treated cells (Table 1). There might, of course, be scenarios in which subtle measurable effects are of interest; however, in the context of large primary screens, such as that conducted by Lawson et al. (2022), this is rarely the case. Note that a similar trend can be observed in this case when considering median values rather than means (Table 1, Fig. 1C overlaid boxplots); in the case of data distributions differing greatly from a normal distribution, comparison of median values might be more appropriate.

However, it is important not to over-interpret this result, as it is based on a single experimental replicate. There is a possibility that any of the selected wells could be outliers and not truly representative of the treatment group due to natural variation or experimental error. Without additional replicates to validate the findings, the results might not be generalisable or reproducible, and conclusions drawn from this single experiment might not hold under slightly different conditions. It is crucial to acknowledge and be aware of this limitation when interpreting results from single experiments – we have no way of knowing whether the result we have observed is the “one chance in a million” (Fisher and Prance, 1974). But let us first examine what happens to the above result if we reduce the number of cells analysed in each well.

### How many cells need to be analysed to accurately describe the population in a given well?

Up to this point, we have only considered results derived from an analysis of all the cells in each well, ranging from 606 to 1362 cells, depending on the treatment (Table 1). We wanted to determine whether similar results could be obtained from analysing a subset of cells from each well. Specifically, we wanted to estimate the smallest sample that could be taken from each well to arrive at a result comparable to that in Table 1.

**Fig. 2.**
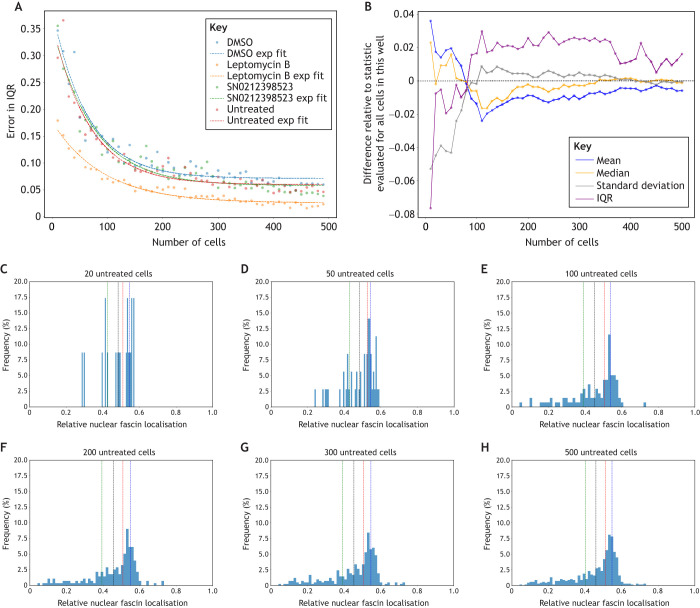
**Variation in statistical descriptors stabilises above a certain sample size.** (A) The difference between the maximum and minimum estimate of the IQR follows a predictable exponential decay as the number of cells in each sample is increased. Results are based on 100 random cell samples for each sample size. Exp fit, fitted decaying exponential function. (B) The difference between the indicated statistical properties of cumulative random cell samples drawn from the population of untreated cells (Table 1) and the properties listed in Table 1 for the entire population. (C–H) Histograms showing the distribution of relative nuclear fascin localisation for the indicated random cell samples as described in B. Vertical lines depict the width of the IQR (green, 25th percentile; blue, 75th percentile), the median (red) and the mean (black).

It is possible to ascertain at what point data collection should cease by examining statistical properties as data are being collected. Indeed, the central limit theorem tells us that there is a predictable relationship between population and sample distribution parameters (Krzywinski and Altman, 2013). We can, for example, consider the interquartile range (IQR), defined as the difference between the 75th and 25th percentiles (see Table 1), a commonly used measure of the spread of a dataset. In Fig. 2A, we show that the uncertainty in this particular statistical descriptor decays approximately exponentially with increasing sample size. In other words, as more data are collected, the error associated with a particular statistical parameter declines in a highly predictable manner.

This is further illustrated in Fig. 2B, which shows how four commonly used statistical parameters describing the untreated cells data (Table 1) vary as a random sample of the data is increased in size. We observe in our example a threshold effect around a sample size of ∼250–300 cells, beyond which additional data yield diminishing returns in terms of statistical insight. This can be seen further in Fig. 2C–H, which show the distribution of data as sample size increases: the distributions for sample sizes of 200 and 500 look very similar.

This evidence suggests a pragmatic approach to data collection: the aim should be to gather enough data to reach statistical stability, where the key metrics of interest no longer change significantly with additional sampling. Once this level of statistical confidence is achieved – often visible through a plateau in the variability of statistical parameters – further data collection becomes less informative and might unnecessarily expend resources. However, it is important to note that this threshold value, at which data collection should cease, is somewhat arbitrary: based on Fig. 2B, we could have decided, not unreasonably, that the statistical descriptors had sufficiently stabilised after analysing 100 cells, for example. The level of precision required will depend on the size of the effect being compared between conditions, which we will discuss in the next section. Ideally, finding this threshold could be achieved via a smart microscopy setup, but we acknowledge that such technology is unlikely to be widely available. Alternatively, see the later section in this Perspective (A framework for robust quantitative bioimaging experimental design) where we discuss how to ‘start small and scale up’.

It is important at this point, to emphasise once again the difference between increasing the number of data points obtained from a single experiment and increasing the number of independent experiments. In the case of the former, as discussed above, drawing additional samples (here, cells) from a single experiment has diminishing returns beyond a certain point – once we have a stable, representative estimate of the mean, for instance, additional data might offer minimal new insight. This differs from collecting independent samples from multiple experimental units, which enhances the generalisability and reliability of the results (Lord et al., 2020). We will revisit this point later in this Perspective.

### How many cells need to be analysed to reliably measure an effect relative to controls?

The number of cells for each treatment in Table 1 is relatively large, and the difference in means is relatively small. However, the data presented in Fig. 2 suggest that we should have reasonably high confidence in the result provided by a random sample of ∼200–300 cells from each treatment group.

**Fig. 3.**
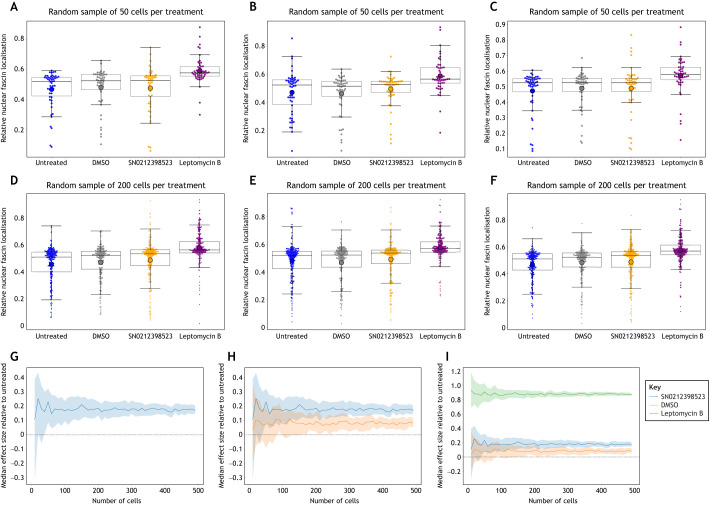
**Effect sizes are relatively insensitive to random sampling and sample size.** (A–F) Three different random samples of 50 (A–C) or 200 (D–F) cells for each treatment. Each small point represents a single cell. The larger dots represent the sample means. Box and whisker plots are overlaid, with the height of the box, the horizontal line within the box and the whiskers representing the IQR, the median and the spread of the data excluding outliers (IQR±1.5×IQR), respectively. (G) The influence of random sampling and sample size on the difference between fascin localisation in SN0212398523-treated cells and that in untreated cells. (H) A repeat of the bootstrapping analysis in G, this time including the effect size of measured nuclear fascin in DMSO-treated cells as a vehicle control, relative to untreated cells. (I) Another repeat of the bootstrapping analysis in G and H, this time including the positive control, leptomycin B treatment. For each sample size in G–I, random sampling was performed 100 times. The solid line represents the median of those 100 random samples, and shaded bands represent the width of the IQR. Effect sizes are calculated according to Eqn 2 (see Supplementary information).

We therefore explored the influence of randomly sub-sampling from each well and examining the impact this has on the results (Fig. 3A–F). For each of these random samples, the distribution shapes might differ, particularly for the smaller sample size of 50 cells (Fig. 3A–C). However, the overall trend in the result is virtually identical: for every random sample, regardless of sample size, the mean (and median) nuclear localisation of the leptomycin B-treated cells is consistently higher than all other treatments. In this case, all experimental conditions stabilise around a similar number (∼200) of analysed cells; if one condition were to require more cells to reach statistical parameter stability, this value should be selected across all treatments.

We further investigated this relationship between sample size (number of cells), random sampling and effect size. We first examined the difference between fascin localisation in untreated cells and cells treated with SN0212398523 (Fig. 3G). For small sample sizes, there is a large amount of uncertainty in the measured effect size (the difference in mean nuclear fascin localisation between the two populations). But, as the sample size increases, the effect of random sampling is reduced, and the result changes minimally. We can reasonably conclude that, in this particular experiment, the measured nuclear fascin localisation is higher in SN0212398523-treated cells than in untreated cells. Adding more cells to the analysis will not alter this conclusion. However, it is not clear how biologically significant this difference is.

We repeated this analysis, but this time included the vehicle control, DMSO-treated cells (Fig. 3H). Above a certain threshold (∼250–300 cells per sample), there is little overlap between the IQRs, and we can reasonably conclude that, according to our analysis pipeline, nuclear fascin localisation is higher in SN0212398523-treated cells than it is in DMSO-treated cells in this experiment. But the fact that Fig. 3H shows that nuclear fascin localisation is also higher in DMSO-treated cells than it is in untreated cells should cause us to question the biological significance of any measured effect of SN0212398523. The higher nuclear fascin levels observed in the presence of SN0212398523 could reflect a genuine biological effect, an influence of DMSO, a technical artefact caused by random well-to-well variability or a combination of these factors. Additional controls are required to aid interpretation.

Repeating the same analysis one more time with a positive control puts these differences into context (Fig. 3I). There are two important points to highlight from these results. The first is that, while there is a measured increase in nuclear fascin localisation in DMSO-treated cells and SN0212398523-treated cells, it does not seem biologically significant, relative to the increase in localisation measured in leptomycin B-treated cells. The second is that, even for small sample sizes (less than 50 cells), this result is apparent. Analysing more cells only serves to refine the measured differences (that is, the uncertainty in the estimates of the means is reduced) – it does not alter the overall conclusion. However, in this particular experiment, the uncertainty in the effect of leptomycin B treatment (the width of the shaded area of the plot in Fig. 3I) is small relative to the effect size (the solid line in Fig. 3I). In other scenarios, in which effect sizes are smaller and the associated uncertainty greater, more data collection might be necessary to draw conclusions from the results.

### Is the relationship between sample size and effect size reproducible across multiple experiments?

There is an important point to be made at this juncture regarding the misuse or misinterpretation of *P*-values. It is not unusual for data similar to that presented in Fig. 1C to be annotated with *P*-values, where each cell is treated as an independent data point (see Table S2). For examples, see figure 1D and E of Cai et al. (2024), or figure 1G–I of Buglak et al. (2024). Without getting into a detailed discussion of what constitutes a technical or biological replicate, it is not unreasonable to state that different cells within the same dish or sample, all subjected to the same treatment, are not replicates (Zweifach, 2024) – they constitute one single experimental unit. Even if each cell could be considered independent, it is well established that *P*-values decay exponentially with increasing sample size (Gómez-de-Mariscal et al., 2021). In other words, with a sufficiently large number of data points, statistical testing becomes redundant, as the null hypothesis will almost always be rejected, even for effects with biologically negligible size. As such, subjecting such data to statistical tests is unlikely to yield additional insights. Regardless, blindly relying on the *P*-values presented in Table S2 might lead us to conclude that SN0212398523 has a highly significant influence on nuclear fascin localisation. The data we have presented up to this point suggest that this is unlikely, but experimental repetition is necessary to confirm.

To test the influence of random sampling and sample size when comparing multiple experiments, we analysed additional data from Lawson et al. (2022) (Fig. S1). We found that increasing the number of cells analysed from 50 to 200 had a small influence on the result (Fig. S1A,B). The distribution of data points changed slightly, but the overall conclusion did not change – leptomycin B has a far larger effect on nuclear fascin localisation than SN0212398523. Consistent with other data presented up to this point, we found that increasing the number of cells in each sample beyond 200 had little effect; we obtained an almost identical result when 500 cells were randomly sampled (Fig. S1C). One could employ an ANOVA test at this point to add additional weight to the result. But given that all box and whisker plots for each treatment overlap, except for those associated with leptomycin B, no statistical test should be necessary to conclude that leptomycin B has a far more significant biological effect than any other treatment. However, a major limitation of the dataset we have relied on thus far is that there is only one single well, or replicate, for each compound tested – very often a feature of preliminary screens. Hence, in Fig. S1, we are comparing one well for SN0212398523 with three for the untreated, DMSO-treated and leptomycin B-treated populations. This is not ideal and gives us a very precisely measured difference between wells, rather than a true inference between treatments. To address this shortcoming and illustrate the robustness of our approach, we now apply the same analysis to a second publicly available dataset.

## Case study 2 – validating our approach with a second dataset

**Fig. 4.**
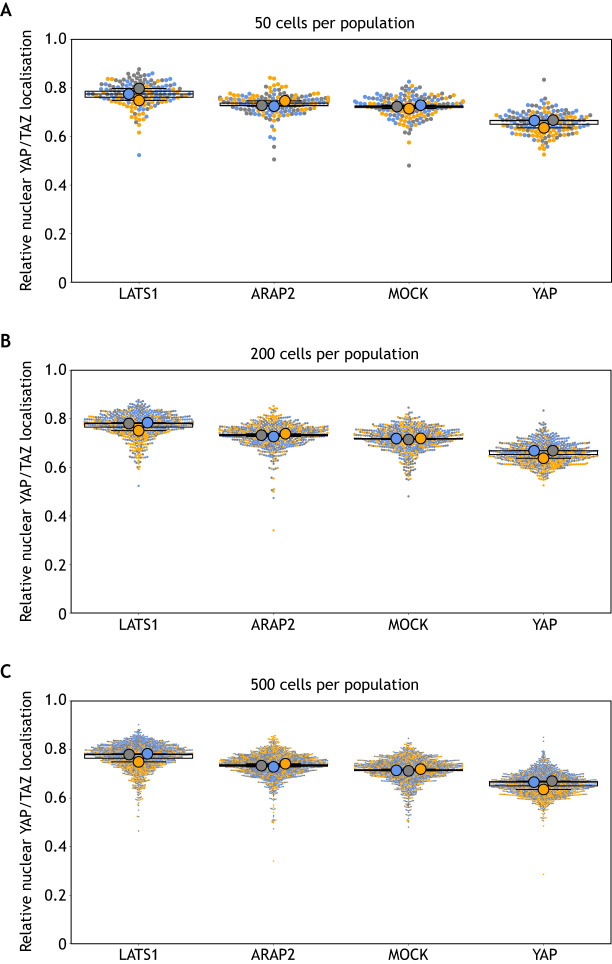
**Effect size is relatively insensitive to random sampling when comparing multiple experiments.** The data are plotted using the ‘superplot’ framework suggested by Lord et al. (2020). (A–C) Nuclear YAP/TAZ localisation averaged across samples of cells subjected to the indicated siRNA knockdown. Each large dot represents the average of 50 (A), 200 (B) or 500 (C) randomly selected cells from three different wells. Each small dot represents a single cell. Dots with the same colour correspond to a particular experimental replicate. Boxes define the IQR of the averages (large dots). The horizontal line within the boxes indicates the median. The whiskers indicate the spread of the data excluding outliers (IQR±1.5×IQR). The source of the data for each replicate is listed in Table S1 – all data are from the same RNAi screen, but wells were selected from three different multi-well plates to simulate experimental repetition.

We reanalysed a subset of the data previously published by Pascual-Vargas et al. (2017) (Fig. 4). The nature of the analysis we conducted is almost identical to that used for the analysis of the data from Lawson et al. (2022), as the images from Pascual-Vargas et al. (2017) also consist of two-dimensional images of cultured cells, labelled with nuclear and actin markers. We again considered the relative localisation of a protein to the nucleus, in this case the transcriptional coactivators YAP and TAZ (also known as YAP1 and WWTR1, respectively; collectively referred to as YAP/TAZ).

Pascual-Vargas et al. (2017) conducted an RNA interference (RNAi) screen using short interfering RNAs (siRNAs) to isolate Rho guanine-nucleotide-exchange factors (RhoGEFs) and Rho GTPase-activating proteins (RhoGAPs) that contribute to morphogenesis in LM2 breast cancer cell lines. Here, we selected the data regarding siRNA knockdown of ARAP2 for reanalysis, comparing it to control siRNA treatments. siRNA knockdown of LATS1 was used as a positive control as it promotes high nuclear YAP/TAZ localisation, and siRNA knockdown of YAP was used as the negative control. We also included a neutral control (mock transfection, vehicle control). We considered three channels in the images, highlighting cell nuclei (stained using Hoechst 33258), YAP/TAZ and actin, once again using the actin channel as a proxy for the cell body. We used the same CellProfiler pipeline as in Case study 1 to quantify the proportion of YAP/TAZ localised to the nuclei under different experimental conditions (see Supplementary information).

### Increased data collection again yields diminishing returns

We begin by demonstrating that the general principles outlined in Fig. 2 for the analysis of data from Lawson et al. (2022) also hold true for that of data from Pascual-Vargas et al. (2017) (Fig. S2). We show again that the uncertainty in the IQR decays exponentially with increasing sample size (Fig. S2A). We go on to show how the mean, median, IQR and standard deviation describing the nuclear YAP/TAZ localisation in mock-transfected cells vary as the size of a random sample of the data increases (Fig. S2B). We again observe a dataset-specific threshold effect, this time around a sample size of ∼200 cells, beyond which additional data yield diminishing returns in terms of statistical insight. We further emphasise this in Fig. S2C–H; the distribution of the data for sample sizes between 200 and 500 looks very similar. However, once again, we emphasise the difference between increasing the number of data points obtained from a single experimental unit and increasing the number of independent experiments (the latter being explored below).

### Effect sizes can be ascertained from small sample sizes

We next explored the influence of random sub-sampling on our analysis of the data from Pascual-Vargas et al. (2017) (Fig. S3A–F). The overall trend in the result is virtually identical for different sample sizes, and the differences in measured effect are similar. Exploring further, we again randomly sampled the data from Pascual-Vargas et al. (2017) and measured the proportion of YAP/TAZ localised in the nucleus across all treatment conditions (Fig. S3G–I). We can reasonably conclude that, in this particular experiment, the nuclear YAP/TAZ localisation will be higher in ARAP2 siRNA-treated cells than in mock-transfected cells (Fig. S3G). We can also conclude that although ARAP2 siRNA-treated cells might exhibit considerably higher nuclear YAP/TAZ localisation than our negative control (YAP RNAi) (Fig. S3H), the effect is not quite as strong as for the positive control (LATS1 RNAi) (Fig. S3I). However, it bears repeating that this is the result of one single experiment, and we cannot reach any firm conclusions until we examine the results of replicates.

### The relationship between sample size and effect size is reproducible across multiple experiments

To test the influence of random sampling and sample size when comparing multiple experimental replicates, we analysed additional data from Pascual-Vargas et al. (2017), incorporating two more wells per treatment drawn from multiple plates. We found that, across a range of different cell sample sizes, the result was highly reproducible (Fig. 4). Increasing the number of cells analysed and averaged per population from 50 to 200 had almost no influence on the result (Fig. 4A,B). The distribution of data points changes slightly with sample size, but the overall conclusion does not change: LATS1 RNAi knockdown has a larger effect on nuclear YAP/TAZ localisation than ARAP2 RNAi knockdown. Consistent with other data presented up to this point, we found that increasing the number of cells in each sample beyond 200 had little effect – we obtained an almost identical result when 500 cells were randomly sampled (Fig. 4C).

## A framework for robust quantitative bioimaging experimental design

Our analysis highlights the risks of over-interpreting small treatment effects, especially without appropriate controls and experimental repeats. Biological significance can only be conclusively inferred when effect sizes are measured against controls that deliver biologically meaningful and measurable effects. Relying solely on arbitrary statistical thresholds, such as *P*-values, to distinguish between significant and insignificant results can lead to misconceptions about the biological importance of findings. We propose several strategies to develop a more nuanced understanding of statistical results and to encourage practices that prioritise biological relevance over arbitrary statistical thresholds when designing imaging experiments (summarised in Box 1).

Box 1. Statistical framework for robust and reproducible bioimage analysis **Pre-experimental planning**

- Outline the entire experimental workflow.
- Explicitly define the metric of interest.

**Small-scale test experiment and determining the dynamic range**

- Use appropriate controls to determine the full range of possible values for your metric.
- Determine the necessary sample size by increasing the number of cells until statistical descriptors stabilise.

**Establishing reproducibility**

- Repeat the small-scale test experiment experiment *n*≥3 times.
- Generate a superplot (Lord et al., 2020) to visualise patterns and trends across all treatments.

**Results and interpretation**

- Prioritise effect size and biological significance over arbitrary *P*-value thresholds.
- Compare treatment effects against the validated range established by your controls.

**Transparent reporting and data sharing**

- Publish image data in a suitable repository, such as the BioImage Archive (Hartley et al., 2022).
- Publish code and pipelines in a suitable repository, such as Github.
- Adhere to FAIR principles (Wilkinson et al., 2016).
- License appropriately (for guidance, see https://focalplane.biologists.com/2023/05/06/if-you-license-it-itll-be-harder-to-steal-it-why-we-should-license-our-work/).

First and foremost, outlining the complete experimental workflow at the outset – from sample preparation to image acquisition, image analysis and data interpretation – is essential. Decisions made at one stage in the experimental process feed forward to affect subsequent steps, and desired endpoints inform how earlier steps should be performed (Senft et al., 2023). So, for example, leaving considerations of image analysis and data interpretation until after sample preparation and image acquisition have been optimised will likely result in a mismatch between the images acquired and the desired result. Although it might seem obvious, discussing the complete workflow with colleagues prior to commencing experimental work, particularly those with significant experience in sample preparation, microscopy and/or image analysis, can provide crucial insights necessary for achieving the desired result.

It is also advisable to involve a statistician in these discussions, if possible. While a statistician might have limited knowledge or insights into sample preparation protocols or microscopy, their advice on data interpretation can strongly influence choices around earlier steps in the experimental workflow. Early collaboration helps with selecting appropriate statistical tests, planning effective experiments and avoiding common pitfalls in *P*-value interpretation. However, we acknowledge that access to dedicated statisticians (or indeed, image analysts) might be limited for many, and much of what follows is written with this in mind.

### Outline the complete workflow for a small-scale experiment

Designing a quantitative imaging experiment for the first time often involves unknowns. Starting with a small-scale experiment to obtain preliminary data and establish criteria for larger experiments is sensible. It is of utmost importance that analysis of the image data is considered from the outset and given equal importance alongside sample preparation and image acquisition. Full interpretation of the resultant data to inform further iterations is essential (Senft et al., 2023). While adopting a ‘start small and scale up’ approach might require additional effort in the short term, it is likely to save significant time and resources over the longer term.

#### Determine the metric of interest

Deciding exactly what is to be quantified as soon as possible is fundamental to the success of a quantitative imaging experiment. The amount of information that can be extracted from microscopy images is vast, even for a relatively small-scale experiment, so establishing precisely which metric is appropriate to answer a specific biological question can be a complex undertaking. Advice on choosing an appropriate metric and the various parameters that can influence it goes beyond the scope of this Perspective; however, excellent guides on the subject already exist (Culley et al., 2024; Fazeli et al., 2025).

#### Determine how many cells are needed per treatment

The number of cells needed for a representative sample depends on the variability in the measured property. Estimating this variability can be challenging, especially with novel cell lines, compounds and imaging systems. However, the code provided with this Perspective (see Supplementary information) can be used to arrive at an estimate of an appropriate sample size. Acquire some images of some cells, analyse them and quantify the metric of interest, then analyse the statistical properties of the population to see how they change as the number of cells is increased. An estimate for a good sample size can be obtained by observing when the statistical descriptors of the distribution stabilise around a constant value and range (for example, as shown in Fig. 2B and Fig. S2B).

#### Estimate the size of the effect to be detected

Repeat the above step for different experimental conditions and determine whether an effect can be detected when comparing treatment versus controls. If not, it could be that additional data are required, that the chosen metric of interest is not suitable, or that there is no measurable effect, and a redesign of the experimental workflow might be required. If an effect is detectable, does it meet the threshold for biological significance? How should such a threshold be calculated? There are no simple answers to these questions, but they must be considered as early as possible to inform further experimental design.

### Estimate the range of the metric of interest using appropriate controls

Experiments often default to the simplest control: comparing treated samples against untreated ones. In such a scenario, it is impossible to determine how biologically significant a measured effect is as there is no frame of reference. The measured effect must be compared to the full range of possible values for the metric of interest. This range can only be established by selecting appropriate positive and negative controls (see Fig. 3G–I and Fig. S3G–I).

What constitutes an appropriate control is highly context dependent, and advice on this matter goes beyond the scope of this article. However, the data presented in this article can guide the reader toward selecting controls that are appropriate for establishing a range for their metric of interest to contextualise treatment effects. Note that, prior to comparing treatments to controls, it is important to ensure that controls are rigorously validated to ensure their effects are reproducible.

#### What if there are no appropriate positive or negative controls?

Depending on the situation, non-biological controls might be appropriate. For example, if measuring colocalisation of punctate proteins, multi-labelled fluorescent beads could be an appropriate positive control, and comparing the signal in one channel with background or an empty field of view might constitute an appropriate negative control.

### How many replicates do I need?

Repeat your initial experiment: are the results reproducible? Produce a superplot (Lord et al., 2020). Is there a clear pattern or trend? If yes, scale up your experiment and attempt to reproduce the result. If the results vary considerably, redesigning the experiment might be necessary.

#### What constitutes a replicate?

A replicate refers to a repeat of the experiment with as many independent factors as possible (e.g. different cells, different wells, different days). There might be limitations related to resources or sample availability, discussed in the next paragraph, but for cells to be considered independent statistical entities, at a minimum they must not be subjected to the same treatment in the same well or dish.

#### What if an experiment cannot be repeated?

There might be circumstances in which it is not practical (or even possible) to repeat a particular experiment. This could be due to limited availability of suitable samples (such as animal or clinical specimens, or primary cell lines, for example), or perhaps insufficient time or funding to conduct experimental repeats. Whatever the reason, it might be necessary to explore alternative, complementary experimental approaches to validate a result. What constitutes a suitable complementary approach will be heavily context dependent, and advice on this goes beyond the scope of this Perspective. However, examples could include using alternative experimental modalities, such as flow cytometry data, to substantiate data from fluorescence microscopy, or using single-cell RNA sequencing data to complement data from spatial omics experiments. In either case, any confounding effects and impacts on generalisability introduced by a lack of repetition should be reported.

### Transparent reporting and data sharing

All image data used in this work were freely shared online by the original authors, allowing us to reuse them. We have made all the code used to generate the figures in this article freely available. We encourage readers to publish their work in a similarly open and accessible manner. We strongly recommend that all bioimage data be published in a suitable repository, such as the BioImage Archive (Hartley et al., 2022), and that all image analysis pipelines and associated code be published in an online repository, such as GitHub and/or Zenodo. We suggest licensing your material appropriately – some guidance on this can be found in a post by Robert Haase on FocalPlane (https://focalplane.biologists.com/2023/05/06/if-you-license-it-itll-be-harder-to-steal-it-why-we-should-license-our-work/). Further guidelines on publishing image analysis pipelines are available elsewhere (Schmied et al., 2024; Miura and Nørrelykke, 2021). As far as possible, all data should meet the FAIR (Findable, Accessible, Interoperable, Reusable) principles for scientific data management and stewardship (Wilkinson et al., 2016).

## Conclusion

Our findings emphasise the importance of using appropriate controls and focusing on effect sizes rather than, for example, focusing solely on *P*-values. By considering biological relevance over arbitrary thresholds of significance, it is possible to derive more robust and reproducible conclusions. These recommendations aim to guide future bioimage analysis toward more meaningful interpretations and efficient experimental designs. More importantly, we stress the use of appropriate controls to discern biological meaningfulness. Although this might introduce greater uncertainty in research outcomes, it is a necessary step toward reducing irreproducible ‘highly significant’ results, thereby advancing the credibility and robustness of scientific research. Our aim is to support the community to move beyond a narrow focus on statistical significance and embrace uncertainty, boosting reproducibility and strengthening the credibility of scientific research.

## Supplementary Material

10.1242/joces.264367_sup1 Supplementary information
