# Supplementary material for: Practical statistics for bioimage analysis – a guide to experimental design and data interpretation
Source: J Cell Sci. 2026 May 27;139(10):jcs264367. doi: 10.1242/jcs.264367 (PMC13286366; doi:10.1242/jcs.264367)
Supplement: Supplementary information [file joces-139-264367-s1.pdf]

Supplementary information

Image data

The image data used in this Perspective, first published by Lawson et al. (2022) and Pascual-Vargas et al. (2017), are freely available on the Image Data Resource (idr.openmicroscopy.org) under accession numbers IDR0028 and IDR0139. A subset of each dataset was used, the details of which are listed in Table S1.

Image analysis

All image analysis was performed with a custom image analysis pipeline implemented in CellProfiler (Stirling et al., 2021), which is freely available on GitHub, along with all other code associated with this study (github.com/FrancisCrickInstitute/Enhancing-Reproducibility). Briefly, the nuclear and actin channels of each image were smoothed using Gaussian filtering. Nuclei were then identified in the smoothed nuclear channel and cell bodies were identified in the smoothed actin channel using minimum cross-entropy thresholding and watershed segmentation. Measurements of the mean intensity of the protein of interest (either fascin or YAP/TAZ) were then made within these segmented objects. The relative nuclear concentration of the protein of interest (L_N) was calculated as the mean nuclear intensity (I_N) divided by the mean cellular intensity (I_C):

$$L_N = \frac{I_N}{I_C} \tag{1}$$

Data analysis

All data analysis was performed in Python using the numpy (Harris et al., 2020), pandas (McKinney, 2010), scikit-posthocs (Terpilowski, 2019) and scipy (Virtanen et al., 2020) packages. Plots were generated using matplotlib (Hunter, 2007) and seaborn (Waskom, 2021). Companion Jupyter notebooks, inclusive of instructions on how to set them up locally using Python environments, are available on GitHub (github.com/FrancisCrickInstitute/Enhancing-Reproducibility).

Journal of Cell Science • Supplementary information

Comparing the error in a statistical parameter of a sample with the size of that sample

To determine the rate at which the range of estimates for a statistical parameter narrows as the number of cells in a sample is increased, we performed a bootstrap-style resampling analysis. For each treatment group and each sample size (ranging from 10 to 500 cells), we randomly sampled (without replacement) the specified number of cells and calculated the IQR of nuclear localisation of the protein of interest. This process was repeated 100 times for each sample size. For each treatment group and sample size, we recorded the minimum and maximum IQR values observed across all iterations. We then used the range of IQR variability (maximum minus minimum) as a measure of error – smaller ranges indicated more stable estimates at that sample size. To characterise the relationship between sample size and estimate stability, we fitted a decaying exponential function to the IQR range data for each treatment group.

Calculating the differences between sample statistical parameters and population parameters

To examine how statistical estimates converge as sample size increases within a single treatment group, we performed a cumulative sampling analysis. We incrementally added cell samples in steps of 10, from 10 up to a maximum of 500 cells. At each sample size increment, we randomly selected additional cells (without replacement) from those not yet included in the cumulative sample. For each cumulative sample, we calculated four summary statistics: the mean, median, standard deviation and IQR. To assess convergence, we calculated the difference between the statistics for each cumulative sample and the corresponding statistic computed using the full dataset for that treatment group. This approach allowed us to estimate the minimum sample size needed for stable, representative estimates of the population parameters, with convergence indicated by differences approaching zero as sample size increased.

Comparing effect sizes to sample sizes

To evaluate the stability and magnitude of treatment effects as a function of sample size, we calculated Cohen’s *d* effect sizes (Cohen, 2013), comparing each treatment group to the control condition across a range of sample sizes. For each predetermined sample size, ranging from 10 to 500 cells, and for each treatment group, we performed 100 independent

Journal of Cell Science • Supplementary information

random samples (without replacement) of the specified size from both the treatment and control groups. For each paired sample, we calculated a standardised effect size (*d*) as the difference between the treatment (x_t) and control (x_c) group means, divided by the standard deviation of the control group (s_c):

$$d = \frac{\overline{x_t} - \overline{x_c}}{s_c} \quad (2)$$

Demonstrating experimental reproducibility with superplots

To account for replicate-level variability and to visualise the hierarchical structure of our experimental design, we generated ‘superplots’ following the framework described by Lord et al. (2020). These plots simultaneously display individual-level data points, replicate-level summaries and overall treatment distributions. For each treatment group, we sub-sampled 50–500 cells from each experimental replicate (without replacement); when no sub-sampling was specified, all available cells were included. Individual cell measurements were displayed as points, colour-coded by replicate identity to visualise within-replicate clustering. For each replicate within each treatment group, we calculated the mean value, which was displayed as a larger point to distinguish it from individual measurements. The overall distribution of replicate means for each treatment was summarised using box plots overlaid on the individual data points.

Journal of Cell Science • Supplementary information

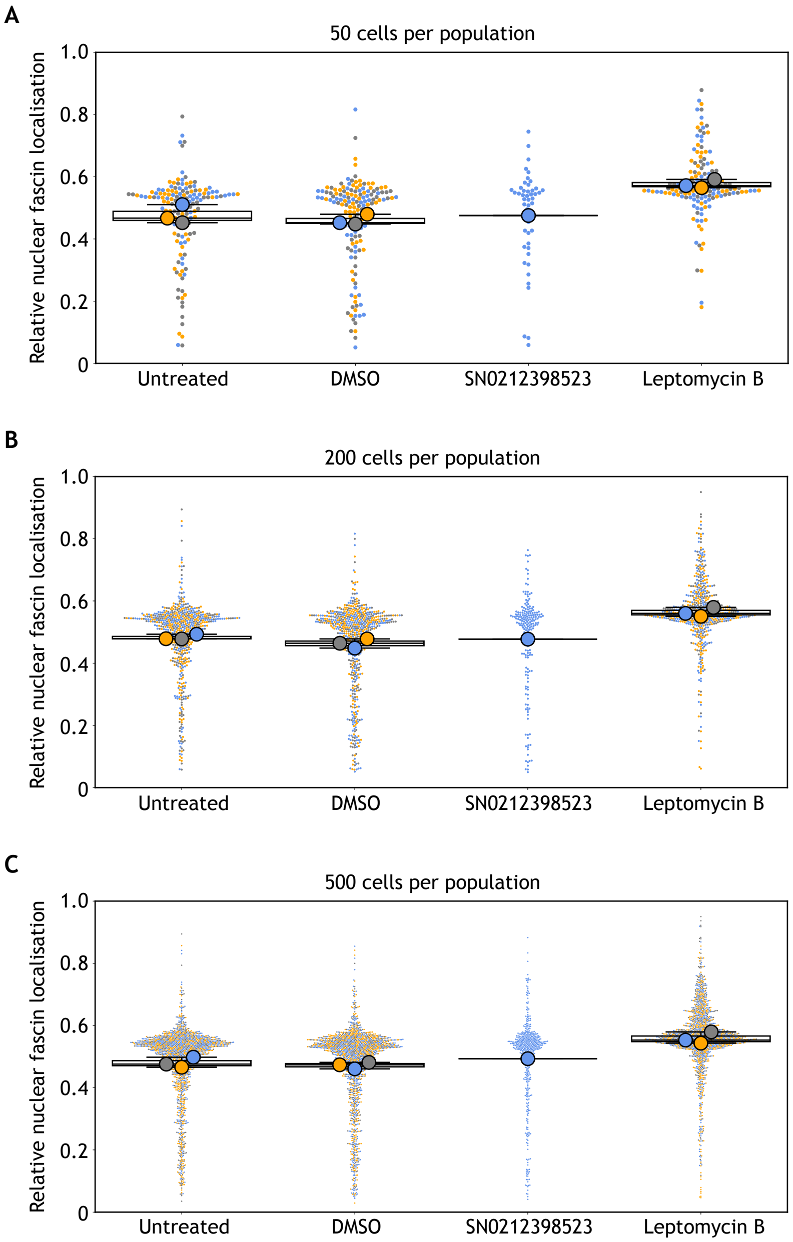

Fig. S1. Effect size is relatively insensitive to random sampling when comparing multiple experiments. The data are plotted using the 'superplot' framework suggested by Lord et al. (2020). (A–C) Nuclear fascin localisation averaged across samples of cells subjected to the indicated treatments. Each large dot represents a technical replicate and is the average of 50 (A), 200 (B) or 500 (C) randomly selected cells within a well. Each small dot represents a single cell. Colours correspond to a particular experimental replicate. Boxes define the IQR of the averages (large dots). The horizontal line within the boxes indicates the median. The whiskers indicate the spread of the data excluding outliers (IQR±1.5×IQR).

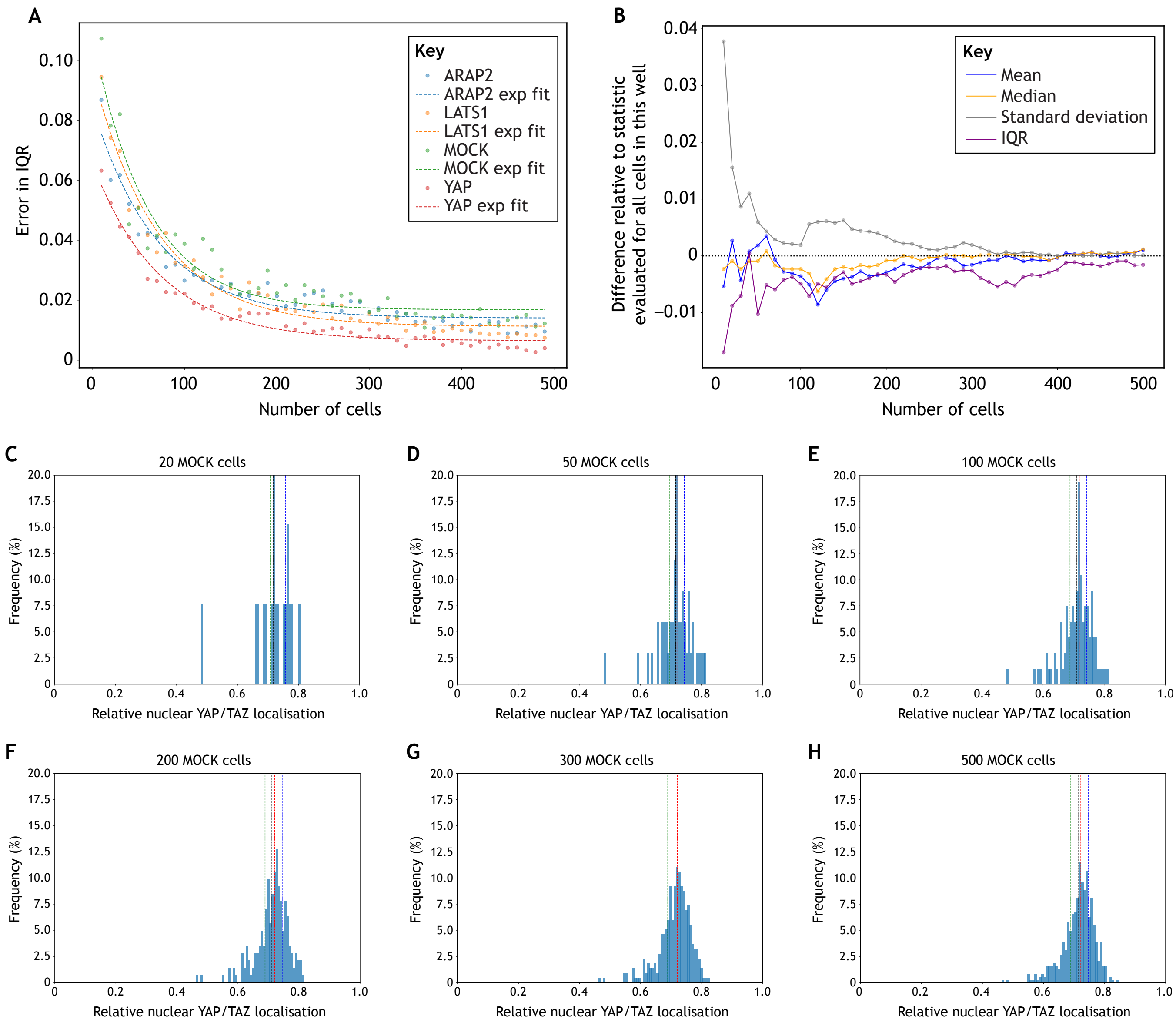

Fig. S2. Variation in statistical descriptors stabilises above a certain sample size. (A) The difference between the maximum and minimum estimate of the IQR follows a predictable exponential decay as the number of cells in each sample is increased. Results are based on 100 random cell samples for each sample size. Exp fit, fitted decaying exponential function. (B) The difference between the indicated statistical properties of cumulative random cell samples drawn from the population of mock-transfected cells (MOCK; see Table S1) and the statistical properties calculated for the entire population. For samples consisting of less than

Journal of Cell Science • Supplementary information

~100 cells, there is substantial variability in the measured statistical properties. However, above a sample size of ~200 cells, the statistical properties are relatively stable and the differences between sample statistics and population statistics tend towards zero. (C–H) Histograms showing the distribution of relative nuclear YAP/TAZ localisation for the indicated random cell samples as described in B. Vertical lines depict the width of the IQR (green, 25th percentile; blue, 75th percentile), the median (red) and the mean (black).

Journal of Cell Science • Supplementary information

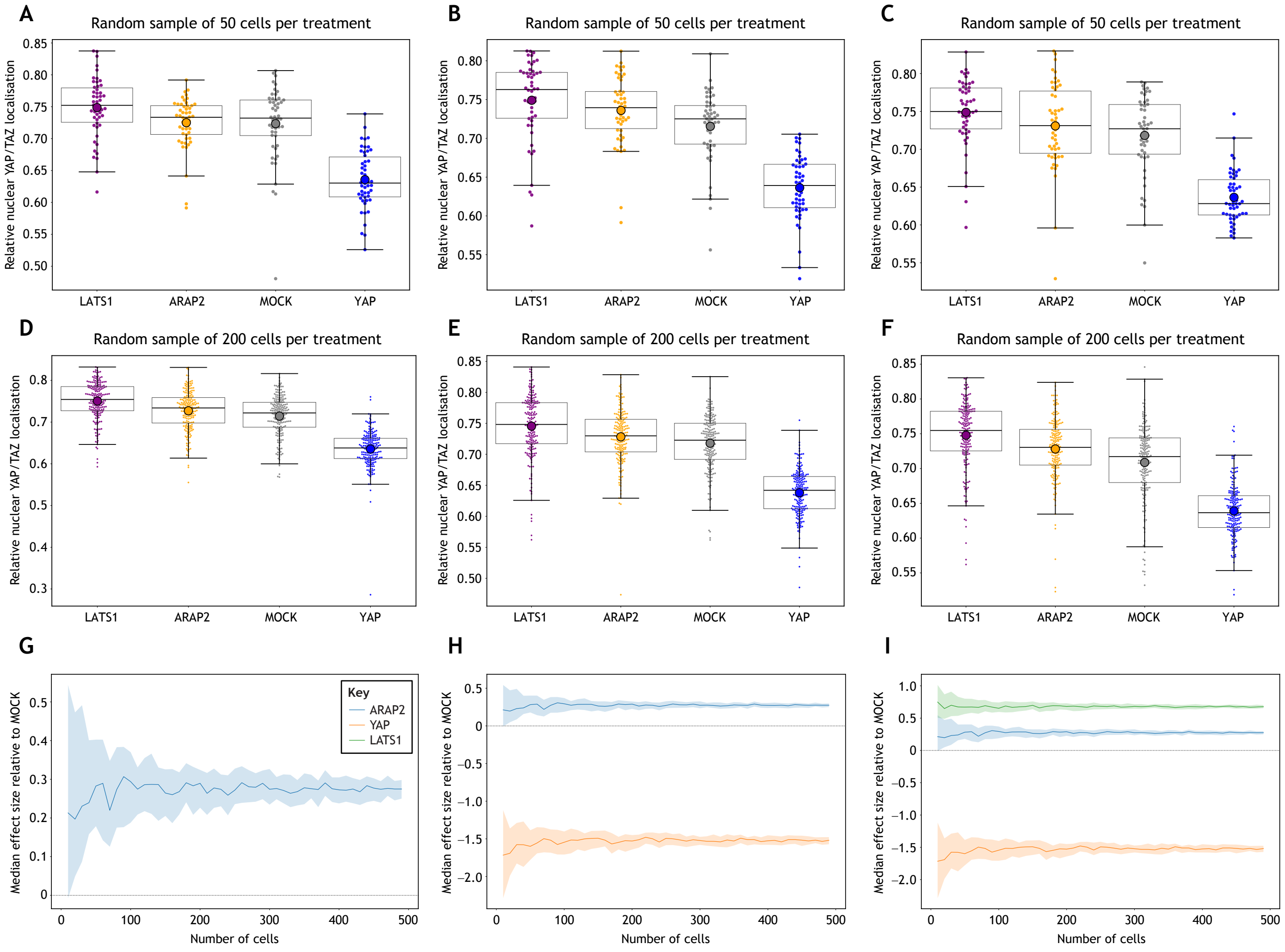

Fig. S3. Effect sizes are relatively insensitive to random sampling and sample size. (A–F) Three different random samples of 50 (A–C) or 200 (D–F) cells for each RNAi treatment (MOCK, mock-transfected cells). Each small point represents a single cell. The larger dots represent the sample means. Box and whisker plots are overlaid, with the height of the box, the horizontal line within the box and the whiskers representing the IQR, the median and the spread of the data excluding outliers (IQR±1.5×IQR), respectively. While the distribution of data points changes in each plot, particularly for the smaller sample size of 50, the overall result is similar in each case. Any random sample of 50 cells or more from these populations will likely show that nuclear YAP/TAZ localisation is higher in LATS1 RNAi cells and lower in YAP RNAi cells. (G) The influence of random sampling and sample size on the difference

between YAP/TAZ localisation in ARAP2 RNAi cells and that in mock-transfected cells. (H) A repeat of the bootstrapping analysis in G, but this time including the effect size of measured nuclear YAP/TAZ in YAP RNAi cells as a negative control, relative to mock-transfected cells. (I) One more repeat of the bootstrapping analysis in G and H, this time including the positive control, LATS1 RNAi. For each sample size in G–I, random sampling was performed 100 times. The solid line represents the median of those 100 random samples, and shaded bands represent the width of the IQR. Effect sizes are calculated according to Eqn 2.

Table S1. Overview of the image data used in this study. All images are publicly available from the Image Data Resource (IDR; idr.openmicroscopy.org; Williams et al., 2017).

Index	IDR accession	Plate	Well	Treatment	Reference
1	idr0139	1093711385	J05	Untreated	Lawson et al. (2022)
2	idr0139	1093711385	I19	Untreated	Lawson et al. (2022)
3	idr0139	1093711385	G15	Untreated	Lawson et al. (2022)
4	idr0139	1093711385	E22	SN0212398523	Lawson et al. (2022)
5	idr0139	1093711385	L08	Leptomycin B	Lawson et al. (2022)
6	idr0139	1093711385	L18	Leptomycin B	Lawson et al. (2022)
7	idr0139	1093711385	H13	Leptomycin B	Lawson et al. (2022)
8	idr0139	1093711385	O02	DMSO	Lawson et al. (2022)
9	idr0139	1093711385	B02	DMSO	Lawson et al. (2022)
10	idr0139	1093711385	N12	DMSO	Lawson et al. (2022)
11	idr0028	LM2 ONTARGETPlus 1A	I23	LATS1 RNAi	Pascual-Vargas et al. (2017)
12	idr0028	LM2 ONTARGETPlus 1A	J23	LATS1 RNAi	Pascual-Vargas et al. (2017)
13	idr0028	LM2 ONTARGETPlus 1A	M23	YAP RNAi	Pascual-Vargas et al. (2017)
14	idr0028	LM2 ONTARGETPlus 1A	N23	YAP RNAi	Pascual-Vargas et al. (2017)
15	idr0028	LM2 ONTARGETPlus 1A	B06	Mock transfection	Pascual-Vargas et al. (2017)
16	idr0028	LM2 ONTARGETPlus 2A	C13	ARAP2 RNAi	Pascual-Vargas et al. (2017)
17	idr0028	LM2 ONTARGETPlus 2A	J23	LATS1 RNAi	Pascual-Vargas et al. (2017)
18	idr0028	LM2 ONTARGETPlus 2A	N23	Mock transfection	Pascual-Vargas et al. (2017)
19	idr0028	LM2 ONTARGETPlus 2A	K16	YAP RNAi	Pascual-Vargas et al. (2017)
20	idr0028	LM2 ONTARGETPlus 2B	C13	ARAP2 RNAi	Pascual-Vargas et al. (2017)
21	idr0028	LM2 ONTARGETPlus 2B	C17	ARAP2 RNAi	Pascual-Vargas et al. (2017)
22	idr0028	LM2 ONTARGETPlus 2B	P13	Mock transfection	Pascual-Vargas et al. (2017)

Journal of Cell Science • Supplementary information

Table S2. *P*-values calculated using a Kruskal–Wallis test followed by Dunn's multiple comparisons test for the data in Fig. 1C. It is inappropriate to use such tests in this case, as the data points (cells in the same wells) are not independent. We highlight this here as it is not uncommon to see statistical tests misused in this way in the literature – for example, see Cai et al. (2024) or Buglak et al. (2024). Because there are so many data points in each distribution, the *P*-values obtained are extremely small, potentially misleading an observer into believing that there are highly significant differences between each population

Comparison	*P*-value
Untreated versus DMSO	<0.001
Untreated versus SN0212398523	<0.0001
Untreated versus leptomycin B	<0.0001
DMSO versus SN0212398523	<0.01
DMSO versus leptomycin B	<0.0001
SN0212398523 versus leptomycin B	<0.0001

Supplementary references

Cohen, J. (2013). *Statistical power analysis for the behavioral sciences*. New York: Routledge. doi:0.4324/9780203771587

Harris, C. R., Millman, K. J., van der Walt, S. J., Gommers, R., Virtanen, P., Cournapeau, D., Wieser, E., Taylor, J., Berg, S., Smith, N. J. et al. (2020). Array programming with NumPy. *Nature* **585**, 357-362. doi:10.1038/s41586-020-2649-2

Hunter, J. D. (2007). Matplotlib: A 2D graphics environment. *Comput. Sci. Eng.* **9**, 90-95. doi: 10.1109/MCSE.2007.55

McKinney, W. (2010). Data structures for statistical computing in Python. In *Proceedings of the 9th Python in Science Conference*. pp. 56-61. doi:10.25080/Majora-92bf1922-00a

Terpilowski, M. A. (2019). scikit-posthocs: Pairwise multiple comparison tests in Python. *J. Open Source Softw.* **4**, 1169. doi:10.21105/joss.01169

Virtanen, P., Gommers, R., Oliphant, T. E., Haberland, M., Reddy, T., Cournapeau, D., Burovski, E., Peterson, P., Weckesser, W., Bright, J. et al. (2020). SciPy 1.0: fundamental algorithms for scientific computing in Python. *Nat. Methods* **17**, 261-272. doi:10.1038/s41592-019-0686-2

Waskom, M. L. (2021). seaborn: statistical data visualization. *J. Open Source Softw.* **6**, 3021. doi:10.21105/joss.03021
